# Assessing hygiene indicators in two dairies in Algeria in producing pasteurized milk

**DOI:** 10.14202/vetworld.2021.2317-2324

**Published:** 2021-09-04

**Authors:** Regguem Souad, Hamdi Taha Mossadak, Bouayad Leila

**Affiliations:** Laboratory of Food Hygiene and Quality Insurance System (HASAQ), Higher National Veterinary School, Rue Issad Abbes, Oued Smar, Algiers 16000, Algeria.

**Keywords:** coliforms, enterobacteria, indicators, pasteurized milk, process hygiene, total microbial flora

## Abstract

**Background and Aim:**

There is a worldwide controversy about the choice of microbial flora for use as process hygiene indicators. This study aimed to evaluate the pertinence of using either coliforms or Enterobacteriaceae (EB) as process hygiene indicators in the pasteurized milk production line. Two flora families and total flora were used as bacterial indicators in some stages of pasteurized milk production line to identify the origin of post-pasteurization contamination and compare the results obtained for each flora. In addition, the bacteriological profile of isolated coliforms and EB was developed.

**Materials and Methods:**

One thousand and two hundred samples of pasteurized cow milk and surfaces (pipes and tank) at various processing stages were taken from two dairies in the northern region of Algeria. The total microbial flora (TF), total coliforms (TC), thermotolerant coliforms, and EB were enumerated, following the recommendations of ISO 4833:2006, ISO 4832:2006, and ISO 21528-2:2017 methods, respectively. The bacteriological profile was determined using the API 20E and 10S tests (bioMérieux, France). Furthermore, the cleaning efficiency and disinfection protocol of surfaces were evaluated using contact agar slides 1 (Liofilchem™, Italy).

**Results:**

Enumeration of the different indicators shows that the highest contamination rate is recorded by the total flora in the two units, 3.28 and 3.78 log CFU/mL, respectively. EB (−0.60 log CFU/mL) at post-pasteurization stage in Unit 1 and coliforms (0.44 log CFU/mL) at the pasteurized packaged milk stage in Unit 2 are the least significant germ families. The lowest compliance rates of bacterial contamination were reported for total flora (82-85%) at the three sampled sites in Unit 2. In comparison, the highest was reported in Unit 1 (99.8%) and 2 (98%) by the EB indicator. Assessing the surface cleaning and disinfection protocol compliance shows that the tank records the highest non-compliance rates for EB and TF (4% and 3%) in Unit 2. EB are represented in both units by various species. *Acinetobacter baumannii* in Unit 1 and *Enterobacter cloacae* in Unit 2 are the common species of the three indicator families. *Acinetobacter* and *Enterobacter* in Unit 1, *Escherichia*, *Citrobacter*, *Enterobacter*, *Klebsiella*, and *Hafnia* in Unit 2 are the most time persistent bacterial genera along the production line. *Stenotrophomonas*, *Serratia, Salmonella, Enterobacter*, and *Escherichia* are common genera in both units.

**Conclusion:**

The results obtained show no difference in the use of EB or TC as hygiene indicators. However, if the objective is to identify the species of bacterial populations, using EBs are the most appropriate.

## Introduction

The dairy industry in developing countries has significant growth potential that is constantly evolving due to the increasing demand for milk and dairy products [1]. Milk provides the human body with all essential amino acids [2]; however, it is unsterile and still contains several microorganisms, which may be pathogenic, causing foodborne illness, nonpathogenic, causing spoilage of the product [3,4]. In addition, because of their composition, milk and dairy products are an excellent growth medium for microorganisms [4,5].

Milk bacterial contamination causes major economic losses and various hazards for human health. More than 20% of milk production in developing countries is lost due to early spoilage and losses due to microbial contamination at different stages of the production [5]. Foodborne diseases caused by pathogenic microorganisms are more frequent than those due to harmful chemicals and plants [6]. The application of heat treatment, such as pasteurization, is sufficient to reduce 99.99% of pathogenic and non-pathogenic microorganisms in raw milk [7]. This treatment eliminates or inactivates all vegetative forms of bacteria, psychrotrophic microorganisms, yeasts and molds, and certain unwanted enzymes while preserving the food value of milk [4,8].

Bacterial contamination of pasteurized milk may have several origins: Manufacturing equipment surfaces, employees’ hands, packaging materials, and deficient pasteurization [9,10]. The latter would allow pathogenic bacteria to survive, leading to production incidents in the post-pasteurization stages (PAST) and subsequently causing health problems for consumers [11,12].

Milk pasteurization has been described as a critical control point (CCP) for implementing food safety management systems [10,11]. Determination and monitoring of CCPs require identifying the origins of the contamination, determination of its persistent nature, and planned corrective actions. Biofilms, the vector of persistent contamination, are bacterial communities that adhere to processing equipment and resist cleaning and disinfection, resulting in continuous contamination of milk and dairy products over time [13]. In addition, the continuous formation of biofilms leads to their resistance to removal, particularly when using cleaning in place systems [14].

An indicator organism is defined as a marker, whose presence reflects, on the one hand, the sanitary status of either a food or an environment, contamination post-application of sanitation treatments, hygienic handling, and storage conditions [14]. However, it can reveal the possible presence of pathogens that are a potential hazard to public health [14,15]. To select the most relevant indicator, it is more appropriate to follow the evolution of several of them over a given period to retain only the one that seems the most sensitive to deviations from hygiene practices [15].

In the dairy industry worldwide, the main groups of indicator bacteria used in post-pasteurization contamination (PPC) are coliforms, Enterobacteriaceae (EB), total Gram-negative, *Pseudomonas*, and Gram-positive spore-forming bacteria [16]. In the US dairy industry, coliforms have been used since 1914 as indicator organisms [17]. They were recommended by the US Public Health Service in the first edition of the Pasteurized Milk Ordinance published in 1924 [6,7]. They continue to be used for this purpose. However, recent studies indicate that only a fraction is of fecal origin, while most would come from the environment [14]. Furthermore, the search for coliforms, which are involved in less than 50% of PPC of milk, does not detect *Pseudomonas* and other Gram-negative non-coliform bacteria [17]. This leads to question about the relevance of using coliforms as hygiene indicators for dairy products [5,14]. In Europe, another widely used group of indicators is proposed for the dairy industry; it is the EB family and the total Gram-negative bacteria [13,16].

This study aimed to evaluate the pertinence of using either coliforms or EBs as process hygiene indicators in the pasteurized milk production line. Two flora families and total flora were used as bacterial indicators in some stages of pasteurized milk production line to identify the origin of post-pasteurization contamination and compare the results obtained for each flora. In addition, the bacteriological profile of isolated coliforms and EBs was developed. There is no Algerian regulatory framework on the indicator to be used to assess the hygiene of processes. The results of this study should provide scientific support to decide on the appropriate indicator to use.

## Materials and Methods

### Ethical approval

The microorganisms studied during the pasteurized milk production process do not require the use of live animals, so no ethical approval is necessary.

### Study period, location, and sample collection

The study was conducted from October 2017 to January 2020. One thousand and two hundred samples were collected from two dairies in the northern region of Algeria. The first one (Unit 1) belonging to the private sector is located in the Wilaya of Tizi-Ouzou; the second (Unit 2) to the public sector is located in the Wilaya of Boumerdès. Twice a week, samples are collected as follows: For each unit, three stages were chosen to collect samples: Immediately after pasteurization (PAST), from the collection tank (Tank), and finally, the pasteurized packaged milk (PPM). One hundred samples of milk and 100 samples of the surface were collected at each selected stage (the surfaces of the tanks by swabbing and the pipes by rinsing). The samples were immediately transferred to the food microbiology laboratory of the National Veterinary School, where they were analyzed on the same day they were collected. The surface samples were taken according to the ISO 18593/2004 method [18].

### Bacteriological analysis of milk

Samples were analyzed for total microbial flora (TF), total coliforms (TC), thermotolerant coliforms (TTC), and enterobacteria (EB) using the following standard methods: ISO 4833: 2006 [19] on plate count agar (PCA); ISO 4832:2006 [20], which includes coliform counts on violet-red bile lactose agar and ISO 21528-2:2017 [21] on violet-red bile glucose agar (VRBG), respectively. In addition, API 20E and 10S strips (bioMérieux, France) were used for bacteriological profile identification. Compliance rates of bacterial contamination for coliforms, total flora, and enterobacteria were evaluated and interpreted according to the Algerian Interministerial Decrees setting microbiological criteria for food products (N35/1998 and N39/2017) [22,23].

### Analysis of surface samples

Contact agar slides with one face to detect EB (VRBG agar) and total flora counting (PCA agar) were used to evaluate the efficiency of cleaning and disinfection protocol of surfaces in contact with milk. The results of the enumeration are given in CFU/cm^2^ of the surface. Compliance is established at 1 CFU/cm^2^ for enterobacteria and 10 CFU/cm^2^ for total flora.

### Statistical analysis

Software R v.3.6.3 (https://www.r-project.org/) was used to analyze the data. Chi-square test and descriptive statistics were used to establish the average and the satisfaction rate. In addition, the ANOVA and Tukey tests were used to compare the compliance rates of the indicators.

## Results

### The overall rate of bacterial contamination of milk

**Figure-1:**
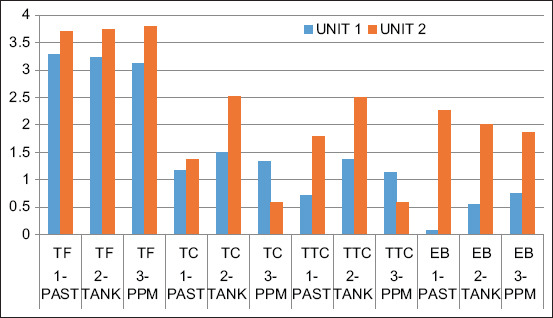
Distribution of the indicators along the production line for both units. Total microbial flora, total coliforms, thermotolerant coliforms, and Enterobacteriaceae.

The enumeration of the different indicator flora shows that the highest contamination rate is recorded by the total flora in the two units, 3.28 and 3.78 log CFU/mL, respectively. The least important germ families are EB (−0.60 log CFU/mL) at the PAST stage (after pasteurization) in Unit 1 and coliforms (0.44 log CFU/mL) at the pasteurized packaged milk stage in Unit 2 (Figure-1). In terms of total and TTC, variable values ranging from 1.13 to 1.48 log CFU/mL and 0.62 and 1.35 log CFU/mL were observed at different sampling sites in Unit 1. However, in Unit 2, tank (2.52 and 2.49 log CFU/mL) and PPM (0.44 log CFU/mL) showed similar mean contamination values as found at both sites, respectively.

### Compliance rate

The highest compliance rates were obtained at the first sampling site (PAST) (99.8% and 98%) in both units using the EB indicator. Conversely, the lowest rates were recorded in Unit 2 by total flora (between 82% and 85%) in all three sites (Table-1).

**Table 1:** Compliance rate of bacterial contamination of milk in both units.

Sample stage	Compliant samples %							

	TC (m=1)		TTC (m=0)		EB (m=M=10)		TF (m=3×10^4^)	
			
	U1	U2	U1	U2	U1	U2	U1	U2
PAST	98	94	98	96	99,8	98	95	85
TANK	94	90	95	94	98.6	94,8	96	84
PPM	96	97	97	97	99.4	97.8	97	82
Mean	96	93.66	96.66	95.66	99.26	96.86	96	83.66

TF=Total microbial flora, TC=Total coliforms, TTC=Thermotolerant coliforms, EB=Enterobacteriaceae, U1=Unit 1, U2=Unit 2; m=Value below which the quality of the product is considered satisfactory, M=Value above which the quality of the product is considered unacceptable, PAST=post-pasteurization stages, TANK=tanker milk, PPM=pasteurized packaged milk

### Evaluation of the efficiency of the cleaning and disinfection protocol

In Unit 1, at the three examined sites, 99% compliance was observed. While, in Unit 2, 4% and 3% of non-compliant samples were reported by EB and TF, respectively, in tank (Table-2).

**Table 2:** Evaluation of the efficiency of the cleaning and disinfection protocol in both units.

Sample stage	TF (C)	EB (C)
	
	›10 cfu/cm^2^	›1 cfu/cm^2^
PAST (U1)	99%	99%
TANK (U1)	99%	99%
PPM (U1)	99%	99%
PAST (U2)	99%	99%
TANK (U2)	97%	96%
PPM (U2)	98%	96%

C=Compliant, TC=Total coliforms, EB=Enterobacteriaceae, PAST=post-pasteurization stages, TANK=tanker milk, PPM=pasteurized packaged milk

### Evolution of the indicators along the process stages

Three combinations of parameters were considered to assess the evolution of the indicators along the production line. Considering the indicator/line parameters, there was no statistically significant difference between the indicators studied during the three stages of the production process (p>0.05) in the two units (Table-3). However, considering the stage/indicator parameters, the difference between stages was significant for all indicators in Unit 1 (p<0.05) compared with Unit 2, which revealed only one difference at the PAST stage at p=0.00133 (Table-4). Finally, a pairwise comparison of the various indicators (indicator/indicator) revealed a significant disparity only when the TF was paired with the other indicators (p=0.0001).

**Table 3:** Assessment of the indicator parameter and production line.

Sample stage	TC		TTC		EB		TF	
				
PAST, TANK, PPM	U1	U2	U1	U2	U1	U2	U1	U2
p-value	0.39	0.51	0.5	0.56	0.5	0.4	0.07	0.32

p=Measure of the probability. TF=Total microbial flora, TC=Total coliforms, TTC=Thermotolerant coliforms, EB=Enterobacteriaceae, PAST=post-pasteurization stages, TANK= tanker milk, PPM= pasteurized packaged milk

**Table 4:** Assessment of the step parameter and indicators.

Indicator	PAST		TANK		PPM	
			
TC, TTC, EB, and TF	U1	U2	U1	U2	U1	U2
p-value	0.0064	0.0013	0.0003	0.114	0.0054	0.70

TF=Total microbial flora, TC=Total coliforms, TTC=Thermotolerant coliforms, EB=Enterobacteriaceae, PAST=post-pasteurization stages, TANK=tanker milk, PPM= pasteurized packaged milk, PPM= pasteurized packaged milk

### Bacterial diversity of hygiene indicators

The identification of different species from different isolated indicator families showed that EB were represented by several species (4) in Unit 1: *Enterobacter cloacae* (Eb cl), *Escherichia coli* 1 (Ec 1), *Salmonella choleraesuis* ssp. *arizonae*, and *Stenotrophomonas maltophilia*; while TC dominated by *Enterobacter aerogenes* (Ebae) and *Serratia odorifera*. The species identified in the TTC family are Ec 1 and *Klebsiella oxytoca* (Klbox).

**Figure-2:**
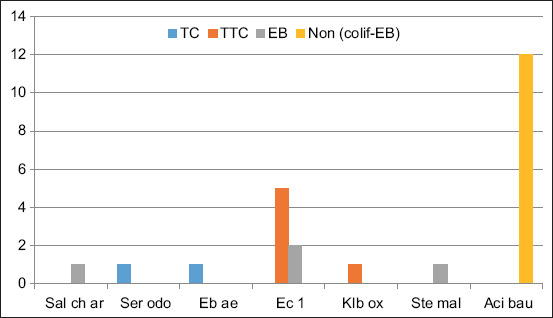
The major bacterial species isolated from the various hygiene indicators in Unit 1. *Acinetobacter baumannii*=Aci bau, *Escherichia coli* 1=EC 1, *Enterobacter aerogenes*=EB ae, *Enterobacter cloacae*=EB cl, *Klebsiella oxytoca*=Klb ox, *Serratia odorifera*=Ser odo, *Stenotrophomonas maltophilia*=Ste mal, *Salmonella choleraesuis* ssp. *arizonae*=Sal ch ar.

**Figure-3:**
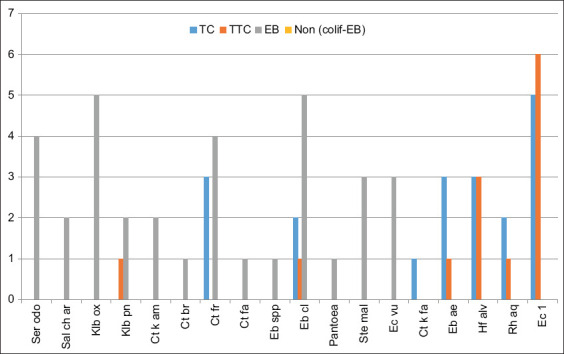
The major bacterial species isolated from the various hygiene indicators in Unit 2. *Escherichia coli* 1=Ec 1, *Enterobacter aerogenes*=Eb ae, *Enterobacter cloacae*=Eb cl, *Enterobacter* spp.=Eb spp., *Citrobacter freundii*=CT fr, *Citrobacter braakii*=Ct br, *Citrobacter farmeri*=Ct fa, *Citrobacter koseri*/*farmeri*=Ct k fa, *Citrobacter koseri*/*amalonaticus*=Ct k am, *Hafnia alvei*=Hf alv, *Rahnella aquatilis*=Rh aq, *Klebsiella pneumoniae* ssp. *pneumoniae*=Klb pn, *Klebsiella oxytoca*=Klb ox, *Serratia odorifera*=Ser odo, *Stenotrophomonas maltophilia*=Ste mal, *Pantoea* spp. 1=*Pantoea*, *Salmonella choleraesuis* ssp. arizonae=Sal ch ar, *Escherichia vulneris*=Ec vu.

Species identification revealed the presence of the non-colif-EB group Acinetobacter. The species with the highest repetition rate is Ec 1 for coliform thermotolerant followed by Eb cl for EB (Figure-2). Figure-3 shows that in Unit 2, 13 bacterial species reported are grouped in the EB family, seven coliforms, and six in TTC. Only one species, Eb cl, is common for all three families (TC, TTC, and EB). However, several species are common in two families: Ec 1, Ebae*, Hafnia alvei*, and *Rahnella aquatilis* for thermotolerant and TC; *Citrobacter freundii* for EB and TC, and *Klebsiella pneumoniae* for EB and TTC. The highest repetition rate was observed in Ec 1(6) followed by Klbox and Eb cl with the same rate (5).

**Figure-4:**
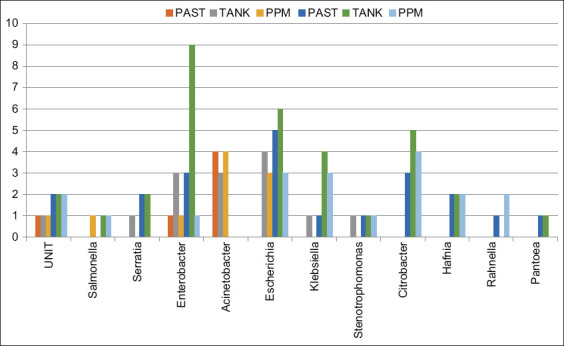
Distribution of Enterobacteriaceae, total coliforms, and thermotolerant coliforms genus during the production process for both units.

The distribution of the established bacterial genera varieties along the production line (Figure-4) shows that *Acinetobacter* and *Enterobacter* are the most persistent genera over time in Unit 1, while *Escherichia*, *Citrobacter*, *Enterobacter*, *Klebsiella*, and *Hafnia* are the most persistent along the process in Unit 2. In both units, we have registered a continuous presence of the genus *Enterobacter* throughout the process. *Stenotrophomonas, Serratia, Salmonella, Enterobacter*, and *Escherichia* are common genera in both units. In addition, *Citrobacter, Hafnia, Rahnella*, and *Pantoea* genera were found in Unit 2 only.

## Discussion

### The overall rate of bacterial contamination

The means of contamination of the total flora reported in Units 1 (3.20 log CFU/mL) and 2 (3.74 log CFU/mL) are similar to those recorded in Cameroon (3.79±0.62 log CFU/mL) [24] and Egypt (3.17 log CFU/mL) [25]. Higher contamination levels, such as those recorded in Kenya (6.05 log CFU/mL) [26] and in Ethiopia (6.60-7.54 log CFU/mL) [27], have been seen in other studies. This could be due to air, packaging, drains, and employees’ contamination [17]. One study found that hygiene practices are insufficient in the entire milk production system in developing countries [28].

The rate of non-compliant samples shown by the total flora in Unit 1 is 4%, and Unit 2 is 16.33%. (Table-1). In Algeria [29,30], rates recorded in recombined milk vary from 0% to 2.17%, respectively. Other reports have found non-compliance rates ranging from 21.4% to 100% [2,26,31,32].

Increased bacterial contamination of treated milk may also lead to inadequate processing procedures, poorly maintained facilities, and staff not trained in hygienic practices. The microbial quality of pasteurized milk is crucially influenced by a high initial concentration of bacteria in raw milk and post-processing contamination [2]. When the initial rate of total flora contamination of the milk tank complies with regulatory requirements, all coliforms are eliminated by pasteurization [33].

The non-compliance rate demonstrated by TC in both dairies ranged from 4% to 6.34%; that of thermotolerant ranged from 3.34% to 4.34%. A similar result (4.8%) was reported in Kenya [26] for TC. Relatively high rates were recorded by Aggad *et al*. [30] (6.52%) and by Hervert *et al*. [17] (7.6-26.6%). Tammam *et al*. [25] (73.3%) and Silva *et al*. [32] registered significantly higher rates (70.8%). Aggad *et al*. [30] observed a non-compliance rate similar to TTC (2.17%) and Silva *et al*. [32] observed a higher rate (57.5%). According to some authors [29,34], the compliance rate could reach 100%, while for another [2], it is the non-compliance rate that can reach 100%. Coliforms do not survive pasteurization but may persist in milk under certain conditions related to pasteurization failures or post-pasteurization recontamination, leading to spoilage or severe foodborne disease [33,35].

The family of EB includes environmental species and other pathogens [36]. The mean levels of EB contamination obtained for the two units (U1 and U2) range from 0.39 to 2.07 log CFU/mL and are lower than those reported by Yilma [27] (3.69 log CFU/mL). The assessment showed that the two hygiene indicator families, EB and TTC, have yielded similar compliance rates.

### Distribution of hygiene indicators in the different stages of production

The results of the assessment of the evolution of the hygiene indicators, considering the parameters of the indicator/line, showed that there was no variance in the two units along the production line (p>0.05); this suggests that the indicator factor does not reveal a significant difference between the different indicator families used.

Taking into consideration the parameters of the step/indicator, a difference was observed in Unit 1 in all stages showing a significant difference between the different production stages and only in the PAST step in Unit 2 (p<0.05), suggesting that each step impacts the production line, illustrating the findings of Eneroth *et al*. [37] who noted that the level of contamination at the PPM packaging stage was higher than at the previous stages and those reported by Aggad *et al*. [38], where the most contaminated is tanker milk (TANK).

Considering the parameters of the indicator/indicator, the findings showed that only the combination of TF with other TC, TTC, and EB indicators created a difference (p=0.0001), confirming the previous hypothesis, which showed that there was no difference in their use as hygiene indicators with the same methods. Therefore, the difference between the impact of the TF and the other indicators makes it a reliable indicator [4].

### Bacterial diversity of hygiene indicators

The study of the bacterial profile of the indicator families used reveals that without excluding coliforms, EB contains a wide range of bacterial genera, including the pathogenic genus *C. freundii*; this supports the results of Ranieri and Boor [39] and Eneroth *et al*. [37]. The genera *Escherichia*, *Klebsiella*, *Citrobacter*, *Enterobacter*, *Serratia*, *Hafnia*, and *Rahnella* represent the coliform family; these findings are similar to those noted by Hervert *et al*. [17].

Previous research has shown that *Acinetobacter baumannii* is a human pathogen that colonizes the skin and upper respiratory tract, suggesting that the contamination found may be of human origin [40]. Using both indicators (coliforms/EB) in both units, the identification of Ec was obtained in the analyzed pasteurized milk, indicating that the contamination is of fecal origin, occurring either during pasteurization failures or during PASTs. Thus, due to fecal contamination during the milking process and poor hygiene practices, *E. coli* can be found in milk and dairy products [41].

Several Ec strains isolated from raw milk and dairy products cause severe foodborne diseases in humans, including hemolytic uremic syndrome, thrombocytopenic purpura, hemorrhagic colitis, and bloody diarrhea [42]. The genera *Klebsiella*, *Enterobacter*, *Serratia*, and *Citrobacter* can originate from feces and environmental sources, making them unreliable as indicators of fecal contamination [43]. Because of their low fecal contamination index, many companies have abandoned total coliform detection for food and water analysis [43].

Some countries support the use of EB as an indicator because of the variety of their isolates, including pathogenic species, such as *Salmonella* spp. and *K. pneumoniae* [17]. Their presence suggests that safety measures are taken during milk processing, and subsequent milk handling has been substandard. Common sources of food contamination by this group of bacteria are feces (animal and human), personal, water, and equipment [44].

The persistence at high levels in the process line of *Acinetobacter*, *Enterobacter*, *Escherichia*, *Citrobacter*, *Klebsiella*, and *Hafnia* indicates that they adhere to milk contact surfaces and is potential persistent or transient colonizers. *A. baumannii* ability to form biofilms on abiotic surfaces makes it possible to grow sustainably in adverse conditions [40].

Defects in the sanitary design of equipment and facilities can create niches where bacteria are protected from disinfectants and survive without biofilm formation [17]. Over time, the development of *Salmonella* is not constant, which suggests that its appearance is accidental and have distinct origins. *Salmonella* can survive for a long period in the environment, more than a year in dust, fuzz, and bovine feces. *Salmonella* spp. can adhere and form biofilms on different materials during their life cycle, and contaminate the food chain, thus representing a potential danger for consumers. Rodents and insects can also be an important source of *Salmonella* [45].

### Controlling the efficiency of the cleaning and disinfection protocol

The control of the cleaning and disinfection process shows a compliance of 99% in Unit 1. Unit 2 showed some limitations, especially in the tank. Nevertheless, the results obtained remain acceptable compared with those published in Albania [9] (13.6% TF and 10.4% EB) and in Macedonia [12] (13.3% TF and 16.6% EB).

Pasteurized liquid milk contamination is due to several factors, including problems with the design of facilities, cleaning and disinfection practices, personal habits, hygiene, plant air control, and cross-contamination [13]. These results confirm the need for regular monitoring of milk stored in tanks.

## Conclusion

The results of this work indicate that the choice of an indicator depends on the objective. For example, if the aim is to perform routine monitoring of the production process, there is no difference in using either indicator (EB/coliforms). However, the ease of culturing coliforms makes them more practical if the objective is to identify the species involved to determine the pathogenicity of the bacterial species and the potential danger to the consumer; the use of EBs remains the most appropriate. Depending on the performance of the heat treatment applied, the multiplication of pathogens is either prevented or stimulated.

As the quality of the products promised to the consumer is often dependent on the control of the finished products, there are many disadvantages to this approach, such as the appearance of food poisoning cases and the increase in the cost of production concerning the recalled non-compliant products. Therefore, it is recommended that the competent authorities generalize the use of process hygiene indicators that allow the efficiency or non-efficiency of manufacturing processes to be verified to avoid multiplying conformity controls of finished products.

## Authors’ Contributions

RS: Performed the study and drafted and revised the manuscript under the supervision of HTM and BL. RS and BL: Interpreted the results. All authors read and approved the final manuscript.
